# The 1000IBD project: multi-omics data of 1000 inflammatory bowel disease patients; data release 1

**DOI:** 10.1186/s12876-018-0917-5

**Published:** 2019-01-08

**Authors:** Floris Imhann, K. J. Van der Velde, R. Barbieri, R. Alberts, M. D. Voskuil, A. Vich Vila, V. Collij, L. M. Spekhorst, Van der Sloot KWJ, V. Peters, H. M. Van Dullemen, M. C. Visschedijk, Festen EAM, M. A. Swertz, G. Dijkstra, R. K. Weersma

**Affiliations:** 1 Department of Gastroenterology and Hepatology, University of Groningen and University Medical Center Groningen, PO Box 30.001, 9700RB Groningen, the Netherlands; 2 Department of Genetics, University of Groningen and University Medical Center Groningen, Groningen, the Netherlands

**Keywords:** Inflammatory bowel disease, Crohn’s disease, Ulcerative colitis, Genome, Microbiome, Transcriptome, Dataset

## Abstract

**Background:**

Inflammatory bowel disease (IBD) is a chronic complex disease of the gastrointestinal tract. Patients with IBD can experience a wide range of symptoms, but the pathophysiological mechanisms that cause these individual differences in clinical presentation remain largely unknown. In consequence, IBD is currently classified into subtypes using clinical characteristics. If we are to develop a more targeted treatment approach, molecular subtypes of IBD need to be discovered that can be used as new drug targets. To achieve this, we need multiple layers of molecular data generated from the *same* IBD patients.

**Construction and content:**

We initiated the 1000IBD project (https://1000ibd.org) to prospectively follow more than 1000 IBD patients from the Northern provinces of the Netherlands. For these patients, we have collected a uniquely large number of phenotypes and generated *multi-omics* profiles. To date, 1215 participants have been enrolled in the project and enrolment is on-going. Phenotype data collected for these participants includes information on dietary and environmental factors, drug responses and adverse drug events. Genome information has been generated using genotyping (*ImmunoChip*, *Global Screening Array* and *HumanExomeChip*) and sequencing (whole exome sequencing and targeted resequencing of IBD susceptibility loci), transcriptome information generated using RNA-sequencing of intestinal biopsies and microbiome information generated using both sequencing of the 16S rRNA gene and whole genome shotgun metagenomic sequencing.

**Utility and discussion:**

All molecular data generated within the 1000IBD project will be shared on the European Genome-Phenome Archive (https://ega-archive.org, accession no: EGAS00001002702). The first data release, detailed in this announcement and released simultaneously with this publication, will contain basic phenotypes for 1215 participants, genotypes of 314 participants and gut microbiome data from stool samples (315 participants) and biopsies (107 participants) generated by tag sequencing the 16S gene. Future releases will comprise many more additional phenotypes and *-omics* data layers. 1000IBD data can be used by other researchers as a replication cohort, a dataset to test new software tools, or a dataset for applying new statistical models.

**Conclusions:**

We report on the establishment and future development of the 1000IBD project: the first comprehensive multi-omics dataset aimed at discovering IBD biomarker profiles and treatment targets.

**Electronic supplementary material:**

The online version of this article (10.1186/s12876-018-0917-5) contains supplementary material, which is available to authorized users.

## Background

Inflammatory bowel disease (IBD), comprising Crohn’s disease (CD) and ulcerative colitis (UC), is a chronic complex disease of the gastrointestinal (GI) tract. IBD is very heterogeneous: disease location within the GI tract, disease behaviour, and average disease activity can vary greatly between patients [1]. Thus far, large numbers of individual genetic variants, environmental factors and gut microbes have been discovered that associate with the onset of IBD [1–9]. However, it remains largely unknown how pathophysiological changes in specific pathways lead to different clinical subphenotypes of IBD, and which treatment could best be applied.

Given our current lack of understanding of these pathophysiological pathways, patients with IBD are classified into subtypes using only clinical characteristics. The classification tool currently in use is the Montreal classification, which consists of age of onset (A), disease location (L), and disease behaviour (B) for CD and age of onset (A), disease extent (E), and disease severity (S) for UC [10]. A combination of the Montreal classification and current disease activity is used to determine a treatment regimen comprising anti-inflammatory drugs and/or surgery.

Meanwhile, the number of new anti-inflammatory biological drugs targeting specific molecular pathways is rising rapidly. Currently, these new drugs are used when regular anti-inflammatory drug treatment fails. However, identifying IBD subtypes on the molecular pathway level could enable a much more targeted treatment approach in which the pathways affected in specific subtypes of IBD could be targeted by specific monoclonal antibodies.

Multiple research groups and consortia throughout the world are generating datasets combining clinical data with either genome data, transcriptome data, or gut microbiome data. By studying these datasets, researchers can answer important individual questions. However, an integrated effort is required if we are to meet the key objectives needed to pursue targeted treatment:

1. To discover molecular subtypes of IBD and match available monoclonal antibody treatment to these subtypes.
2. To discover biomarker profiles that capture the clinical heterogeneity of IBD and can be used as predictors.
3. To discover and prioritise the best new targets for early-stage drug discovery.

Meeting these objectives requires the assessment and integration of different layers of -*omics* information, supplemented with high-resolution phenotype data in the *same* IBD patients. Important IBD multi-omics projects already exist in the longitudinal integrative Human Microbiome Project [11] (iHMP or HMP2), the PRISM-cohort [12], and the RISK-cohort [7] in the United States. Previous efforts from the RISK cohort, the PRISM cohort, and samples from patients treated in our university hospital, as well as from consortia in which these cohorts participate, have already enabled the first steps towards precision medicine in IBD. For example, the discovery of genetic variants enables the prediction of the risk of pancreatitis as a severe side-effect of azathioprine, a commonly used immunosuppressant in IBD [13]. Microbial DNA profiles and RNA-sequencing profiles from the intestinal biopsies of the RISK cohort have uncovered RNA-microbe interactions and shown that biopsies taken from the distal colon can predict the IBD disease location higher up in the intestine [7, 14]. In addition, stool samples from the PRISM cohort have also been used to discover microbial profiles that can predict the efficacy of vedolizumab, a biological drug regulating T-cell homing to the gut [15], while a genetic variant in the WWOX gene discovered using genotypes of IBD patients treated in our hospital can be used to assess the risk of stricturing and penetrating Crohn’s disease behavior [16].

We initiated the 1000IBD project to prospectively follow more than 1000 IBD patients from the Northern provinces of the Netherlands and collect a uniquely large number of phenotypes. Phenotype data include—but are not limited to—information on dietary and environmental factors, drug responses and adverse drug events. In addition, we will generate cross-sectional multi-omics layers.

Here, we report on the establishment and future development of the 1000IBD project and release the first data from the project. This first release and future data releases will be stored externally in the European Genome-phenome Archive (EGA) of the European Bioinformatics Institute (EBI) and Centre for Genomic Regulation [17]. This first 1000IBD data release includes basic phenotypes of 1215 participants, host genotypes based on the Immunochip for 314 participants, and gut microbiome data from 315 stool samples (one per participant) and 107 biopsies (one per participant) generated by tag sequencing the 16S rRNA gene.

## Construction and content

To create the 1000IBD dataset, we collected and generated extensive prospective phenotype, diet and environment data, extensive treatment response and adverse treatment event data, and a *multi-omics* dataset of the same patients.

### 1000IBD cohort, patient selection and recruitment

All IBD patients treated in the specialized IBD Center of the Department of Gastroenterology and Hepatology of the University Medical Center Groningen (UMCG) are asked to participate in the 1000IBD project. Patients recruited for the 1000IBD project include new patients with new-onset IBD, new patients with existing IBD newly referred to our hospital, and patients with IBD already being treated in our hospital. Since there are more than 2000 IBD patients already being treated in our hospital, this last group is gradually being asked to participate to allow our research nurses to obtain the data and samples in an organized way. Since our university hospital is a tertiary referral centre, the number of new-onset IBD patients is limited.

The only inclusion criteria for the 1000IBD project are that patients need to: (1) be at least 18 years old, (2) have IBD based on accepted radiological, laboratory and endoscopic findings, (3) have provided informed consent and (4) able to speak, read and write Dutch in order to be able to fill in the questionnaires. Paediatric patients are not included in 1000IBD because our institutional review board (IRB) approval does not cover them. There are no additional inclusion or exclusion criteria. Inclusion is still on-going, and the project had enrolled 1215 IBD patients as of September 1, 2017.

### 1000IBD informed consent and IRB approval

All patients are asked to sign an informed consent form. Once they have given written informed consent, we collect, generate and integrate clinical data, diet and environmental data, genome data, transcriptome data, and microbiome data for each participant. The 1000IBD project was approved by the UMCG IRB (IRB number 2008.338).

### 1000IBD: Logo

**Fig. 1:**
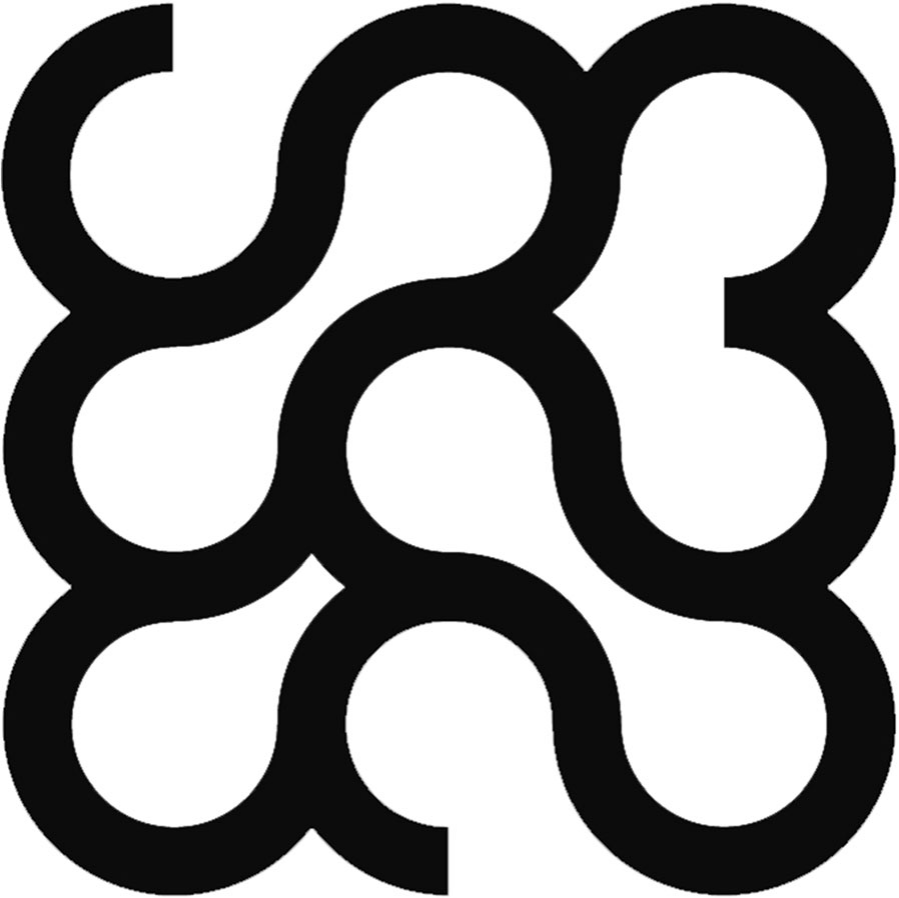
1000IBD Project Logo. This logo depicts the intestine and the multifaceted character of the project

A 1000IBD logo depicting the intestine and the multifaceted character of the project was created to represent the project (Fig. 1).

### 1000IBD Datamodel and identifier

**Fig. 2:**
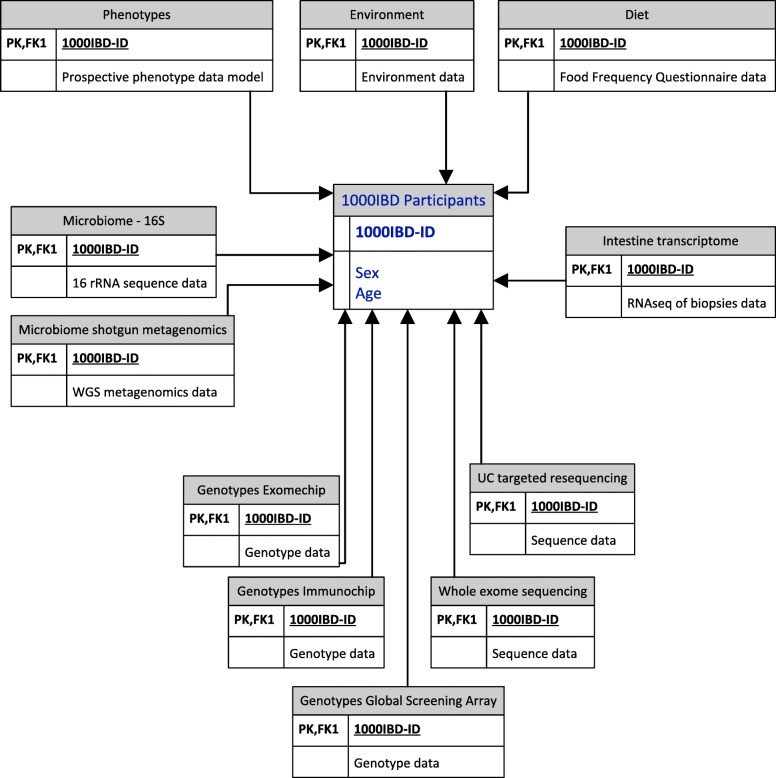
Simplified 1000IBD data model. 1000IBD-ID is the 1000IBD identifier used in every data-layer, also referred to as primary key (PK) and foreign key 1 (FK1). RNAseq: RNA-sequencing, 16S: Sequencing data of the microbial 16S rRNA gene; WGS: whole genome shotgun sequencing

Every 1000IBD participant has a unique pseudo-anonymized 1000IBD-identifier that is used to link all clinical phenotypes and molecular data layers. A model of the 1000IBD dataset is depicted in Fig. 2.

### Generation of clinical phenotype and treatment response data

The collection of prospective data on clinical phenotypes, treatment responses and adverse events is incorporated into the regular IBD clinical care process in the outpatient department of our IBD centre. These phenotype and treatment data serve both as the electronic health record and as a research dataset. This approach of collecting data ‘at the source’, i.e. during the patient visit, has been recommended by the Dutch Federation of University Medical Centres (NFU) [18].

An extensive prospective phenotype data model was developed to ensure uniform data collection over time and across different healthcare professionals. Data items and descriptions of the data model are listed in Additional file 1: Table S1. This phenotype model is similar to the phenotype model of the *Dutch IBD Biobank* (part of *Parelsnoer* [19]) to which a subset of the 1000IBD phenotypes is automatically uploaded [20]. At inclusion, the research nurse fills in most of the data items. The gastroenterologist subsequently only updates data items that have changed since the last patient visit. Table 1 provides an overview of the available data on the most important phenotypes and summary statistics of the 1215 1000IBD participants.

**Table 1:** Clinical phenotypes of 1215 1000IBD participants

No. of participants	1215
Age (Median ± IQR)	41 ± 25
Sex	
Male (%)	510 (41.97)
Female (%)	705 (58.03)
Diagnosis	
Crohn’s Disease (%)	615 (50.62)
Ulcerative Colitis (%)	495 (40.74)
IBDU (%)	61 (5.02)
Other (microscopic colitis, IBDI, reconsidering IBD diagnosis) (%)	44 (3.62)
Montreal Classification	
A: Age of Onset	
A1 (%)	159 (13.09)
A2 (%)	710 (58.44)
A3 (%)	253 (20.82)
L: Disease Location (CD only)	
L1 (%)	224 (36.42)
L2 (%)	120 (19.51)
L3 (%)	255 (41.46)
L4 (%)	65 (10.57)
B: Disease Behaviour (CD only)	
B1 (%)	301 (48.94)
B2 (%)	208 (33.82)
B3 (%)	102 (16.58)
Perianal	189 (30.73)
E: Disease Extent (UC only)	
E1 (%)	57 (10.25)
E2 (%)	162 (29.13)
E3 (%)	299 (53.78)
S: Disease Severity (UC only)	
S1 (%)	29 (5.22)
S2 (%)	139 (25.00)
S3 (%)	191 (34.35)
S4 (%)	119 (21.40)
Age at Diagnosis in years (Median ± IQR)	27 ± 19
Disease Duration at Recruitment in years (Median ± IQR)	8 ± 12
Medication Exposure	
Steroids %	90.07
Steroids CD %	91.99
Steroids UC %	88.28
Steroids IBDU %	88.33
Immunosuppressors %	68.32
Immunosuppressors CD %	79.08
Immunosuppressors UC %	56.97
Immunosuppressors IBDU %	56.67
Biologicals %	37.30
Biologicals CD %	55.07
Biologicals UC %	17.37
Biologicals IBDU %	25.00
Mesalazines %	44.34
Mesalazines CD %	18.06
Mesalazines UC %	70.99
Mesalazines IBDU %	83.33
Average Disease Activity^a^	
HBI (Average ± Standard Deviation)	2.99 ± 3.18
SSCAI (Average ± Standard Deviation)	1.61 ± 1.97

^a^For each patient, the median disease activity was determined. For the entire group the average of the individual medians is presented here

*IQR* interquartile range, *CD* Crohn’s disease, *UC* ulcerative colitis, *IBDU* inflammatory bowel disease undetermined, *IBDI* inflammatory bowel disease intermediate, *HBI* Harvey-Bradshaw Index, *SSCAI* Simple Clinical Colitis Activity Index

### Generation of dietary and environmental data

We developed two new questionnaires to gather dietary and environmental data of IBD patients. 1000IBD participants can fill out these questionnaires using a secure web application.

The Groningen IBD-specific Food Frequency Questionnaire (GrIB FFQ) was designed to assess the current dietary habits and nutritional intake of IBD patients. It consists of 119 questions on food items that are grouped into categories: breakfast, lunch, dinner, snacks and drinks. Since IBD patients often follow unguided dietary habits, i.e. those made without consulting a physician or dietician first, population-specific and more extensive items (e.g. dairy substitutes, meat replacers and supplements) are included in the questionnaire. When using this nutritional tool, patients report the intake of foods consumed during the previous month. The food data obtained via the GrIB FFQ will be converted into energy and nutrient intake (in grams/day) using the NEVO food composition database of 2016 (﻿NEVO 2016, RIVM, Bilthoven, the Netherlands). The nutritional intake part of the GrIB FFQ was developed in collaboration with, and validated by, the division of Human Nutrition of Wageningen University using standardized procedures [21, 22].

The GrIB FFQ provides a broader overview than traditional food questionnaires. It also assesses factors that influence nutrition expenditure but are often disregarded. To complement the questions on nutritional intake, items on patient’s conceptions about the role of nutrition in IBD have been added. Since these additional items could not be included in the standard validation procedure of the Wageningen University, the entire GrIB FFQ will be validated with data collected in an upcoming randomized controlled trial.

The Groningen IBD Environmental Questionnaire (GIEQ) is designed to study the role of lifestyle and environment in the development and course of IBD. The GIEQ includes a large number of factors that could potentially influence disease risk and disease course such as mode of birth (vaginal vs. caesarean), whether patients were breastfed, their living surroundings and sun exposure. In the GIEQ, 1000IBD participants are also asked about differences in lifestyle before and after their IBD diagnosis to see which lifestyle changes coincide with the onset of IBD. The GIEQ has just been published and validated [23].

### Generation of new molecular data and previously generated data

Molecular data of multiple –omics layers is being generated during the 1000IBD project. However, the 1000IBD cohort is comprised of both newly generated molecular data and data that was previously generated from samples from the same IBD patients. If informed consent was given, all this data has been added to the 1000IBD project.

### Generation of host genetic data

Host genomic data is generated in the 1000IBD project using genotyping (Illumina *ImmunoChip, Global Screening Array,* and the *HumanExomeChip*) and sequencing (*Pooled targeted re-sequencing* and *whole exome sequencing)*. Peripheral blood samples were drawn from 1000IBD participants and DNA was isolated from EDTA stabilized blood using either phenol-chloroform or the Qiagen Autopure LS with Puregene chemistry (Qiagen, USA), as previously described [24].

#### Genotyping using the ImmunoChip

Host DNA samples from 314 of the 1215 1000IBD participants was genotyped using the ImmunoChip [25] during previous projects between 2010 and 2013 [3], and this data has been added to the 1000IBD project. The ImmunoChip is an Illumina Infinium array comprising 196,524 Single Nucleotide Variants (SNVs) as well as a small number of insertion and deletion markers. These SNVs and markers were selected based on results from genome-wide association studies of IBD and 11 other immune-mediated diseases. Normalized intensities for all samples were called using the OptiCall clustering program [26]. Marker and sample quality control was performed as described previously [6]. Because the ImmunoChip is no longer available, no additional DNA samples will be genotyped by this method. Newer arrays such as the Global Screening Array (GSA) will now be used to ensure that all 1000IBD participants are genotyped.

#### Genotyping using the GSA

At the moment, host DNA samples from 1013 of the 1215 1000IBD participants have been genotyped using the Infinium GSA-24 v1.0 BeadChip combined with the optional Multi-Disease drop-in panel (GSA-MD). The GSA-MD array comprises a multi-ethnic genome-wide backbone combined with Multi-Disease Drop-in content derived from exome sequencing and meta-analyses of several phenotype-specific consortia including the International IBD Genetics Consortium (IIBDGC). The GSA-MD includes over 700,000 genetic variants. Genotypes were called using the OptiCall [26] clustering program (opticall.bitbucket.io) and quality control steps were performed using PLINK 1.9 (www.cog-genomics.org/plink/1.9/) [27]. The remaining 1000IBD participants, as well as future 1000IBD participants, will also be genotyped using the GSA.

#### Genotyping using the Illumina HumanExomeChip

DNA samples of 419 CD patients were genotyped using the Illumina HumanExome-12 v1.1 BeadChip array, which contains 242,901 exonic genetic variants, the majority being low-frequency or rare non-synonymous, splice, or stop-altering variants. The remaining 1000IBD participants will not be genotyped using the HumanExomeChip, but will instead be sequenced using Whole Exome Sequencing.

#### Pooled targeted re-sequencing of UC patients

Host DNA samples of pooled targeted deep high-throughput sequencing has been performed of 122 UC-associated genes [28]. Pooled targeted enrichment of DNA from 404 1000IBD participants (12 individuals per pool) was performed using a custom-made kit (Agilent HaloPlex, designed with Agilent’s Sure Design), resulting in coverage of 99.9% of the target sequence. After enrichment, sequencing was performed on the Illumina HiSeq 2500.

#### Whole exome sequencing

Host DNA samples from 1003 1000IBD participants were whole exome sequenced according to the Broad Institute of Harvard and MIT Standard Human Whole Exome Sequencing v5 (http://genomics.broadinstitute.org/products/whole-exome-sequencing). DNA samples were processed using the Illumina Nextera preparation kit and hybrid capture was performed using Illumina Rapid Capture Enrichment (37 Mb target). Sequencing was done on the Illumina HiSeq platform to generate 150 bp paired DNA reads. Exome sequencing reads were subsequently processed in accordance with the Broad Institute of Harvard and MIT best practice guidelines (https://software.broadinstitute.org/gatk/best-practices/). Reads were mapped to the human genome reference sequence build 37 using BWA MEM5. The Genome Analysis Toolkit (GATK) [29] version 26 was used to call alleles at variant sites as previously described [30]. The VQSR pipeline was used to assess the quality of called variants.

### Intestinal biopsies and the generation of transcriptome data

Intestinal biopsies were collected from 1000IBD participants during colonoscopies for regular clinical care. These biopsies were immediately snap-frozen by the endoscopy nurse or research technician present during the endoscopy procedure using liquid nitrogen, which is readily available in the endoscopy room. We were able to successfully isolate both DNA and RNA from the snap-frozen samples, and results using the bacterial DNA from biopsies have already been published [31]. To date, 5933 biopsies from 900 patients have been snap frozen in liquid nitrogen and stored at − 80 °C. DNA and RNA were simultaneously isolated from 300 fresh frozen human intestine biopsies using the AllPrep DNA/RNA Mini kit (Qiagen, REF no: 80204) according to the company’s protocol. Biopsies were homogenized in RLT plus buffer containing β-mercaptoethanol using the Qiagen Tissue Lyser with stainless steel beads (5 mm mean diameter, Qiagen REF nr: 69989). Sample preparation was executed using the BioScientific NextFlex mRNA sample preparation kit. Sequencing was performed on the Illumina NextSeq500 sequencer. The RNA samples were pseudo-randomized on plates to assure that no single factor was dominant on one plate (IBD diagnosis, disease location or disease activity). RNA-sequencing was conducted in two batches comprising one pilot batch of one plate of 20 samples, and one batch of one plate of 80 samples and two plates containing 100 samples each. When a principal coordinate analysis was executed as part of the QC, no relevant batch effect was detected. The 300 samples generated 20 million reads per sample.

### Generation of gut microbiome data

To date, stool samples have been collected from 544 participants of 1000IBD. Participants were asked to freeze a stool sample within 15 min of stool production at home. A medical student or research nurse visited each participant at home shortly after production to collect the sample on dry ice for transport to the laboratory at -80 °C. We surveyed 248 IBD patients who took part in the stool sampling. Of these 248 patients, only 3 required more than 15 min to store their faecal sample in their freezer and 13 did not fill in this particular question. (Bolte et al. Submitted).

In the laboratory, microbial DNA was isolated using the Qiagen AllPrep DNA/RNA Mini Kit cat. # 80204 with the addition of mechanical lysis, as previously described [7]. The microbial DNA samples were randomized on 96-well plates so that age and IBD diagnosis were mixed. The plates containing microbial DNA were sent to the Broad Institute, Boston, USA for 16S sequencing in one batch. These microbial DNA samples were stored at the Broad Institute at -80 °C, and whole genome metagenomic sequencing was performed at a later stage. A second batch of microbial DNA was sent to the Broad Institute for whole genome shotgun metagenomic sequencing using the same procedure. When a principal coordinate analysis (PCA) was executed as part of the QC, no relevant batch effects were detected.

#### 16S rRNA gene tag sequencing data

The hyper-variable region V4 of the 16S rRNA gene of microbial DNA of 315 stool samples from 315 1000IBD participants and 107 intestinal biopsies of 107 1000IBD participants was sequenced using the Illumina MiSeq. After sequencing, custom scripts were used to remove the primer sequences and align the paired end reads [7].

#### Shotgun metagenomic sequencing data from stool samples

Microbial DNA of 544 stool samples from 544 1000IBD participants was whole genome shotgun sequenced using the Illumina HiSeq platform. Basic QC was performed by the sequencing facility using an in-house pipeline to remove low quality reads from the raw metagenomic sequencing data. Samples with a read depth less than 10 million reads were excluded. Next, quality trimming and adapter removal was performed using Trimmomatic (v.0.32) [32].

### Future LONGITUDAL sampling of biomaterial

In addition to the current 1000IBD biomaterial collections, subsets of the 1000IBD cohort will be sampled longitudinally. In the IBD Tracker project, a selection of 1000IBD participants will undergo weekly stool sampling. A separate IRB approval has been obtained for this project.

### Local infrastructure and software

#### Structured IBD-specific electronic health record

All clinical phenotype data from described in the information model (Additional file 1: Table S1) is collected using an IBD-specific electronic health record (IBD-EHR). This IBD-EHR is used in clinical care and will, in time, be integrated into the new UMCG hospital electronic health record system, EPIC (http://www.epic.com).

#### Patient reported outcome measures using an online tool: myIBDcoach

Some phenotypes are scored by the IBD patients themselves. To assist patients in scoring these patient-reported outcome measures (PROMS), e.g. clinical disease activity, a smartphone *app* and a web application called *myIBDcoach* is used [33]. Gradually, more PROMS will be implemented and added to the phenotype model.

#### Research data in MOLGENIS research

Once phenotype data is extracted from the IBD- EHR and the myIBDCoach smartphone app, it is uploaded into *MOLGENIS Research*, a web-based application that automatically generates the summary statistics [34, 35].

#### Raw sequencing data stored on a high-performance computer cluster

Raw sequencing data is stored on the Calculon high-performance computer cluster of the UMCG.

#### Privacy and information security

The software and information infrastructure were built taking into account Dutch NEN7510 [36] and international ISO 27.001 [37] information security guidelines as much as possible. A penetration test will soon be performed by the UMCG IT department on https://1000IBD.org.

## Utility and discussion

### Use of 1000IBD data

The data of the 1000IBD project has already led to a number of important discoveries. The Immunochip genotypes were used to unravel the host genetic landscape of IBD as part of the IBD Genetics Consortium effort [2, 3, 6]. The UC pooled targeted resequencing study lead to the discovery of a novel UC-associated variant [28]. The gut microbiome data was used to detect the microbial composition of the gut in IBD patients [8]. Phenotype, genotype and gut microbiome data were integrated to look at the complex relations between these different data layers [8, 31].

The 1000IBD data is a versatile resource. Researchers investigating IBD can use the data as a replication cohort or as a pilot cohort to test new hypotheses. Software developers can use the data to test new tools, for example those built to analyse the genome or the gut microbiome. Mathematicians and statisticians can apply new statistical models to the data. A major advantage of the 1000IBD data for all these researchers is that they will not have the considerable cost of building a cohort or of generating genotype, whole exome, transcriptome and gut microbiome data.

### Sharing 1000IBD data in releases

**Fig. 3:**
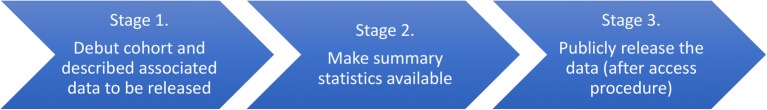
Flow of research data from the 1000IBD project. In Stage 1, data that has been generated or will be generated is announced. In Stage 2, summary statistics will be made available. In Stage 3, the data itself will be publicly released

The 1000IBD data will be made available in three stages (Fig. 3). In Stage 1, data that has been generated or will be generated is announced. In Stage 2, summary statistics will be made available. In Stage 3, the data itself will be publicly released. In the end, all data that does not violate patient privacy regulations will be made publicly available.

### Data release 1

The first release of 1000IBD contains the basic phenotypes of 1215 participants, Immunochip genotypes of 314 participants and gut microbiome data of 315 participants. The content of the data release 1 is presented in Table 2.

**Table 2:** Content of Data release 1. Released on June 5th, 2018

Data	Available for number of participants	Format available
Clinical phenotypes • Age in years • Sex • BMI • IBD diagnosis • Montreal classification: o A: Age of onset o *L: Disease location (CD only)* o *B: Disease behaviour (CD only)* o *E: Disease extent (UC only)* o *S: Disease severity (UC only)*	1215 of 1215 participants of 1000IBD -omics data available from 557 of the 1215 participants of 1000IBD	TSV (Tab-separated file)
Genome		
-Immunochip genotypes	314 of 1215 participants of 1000IBD	IDAT
Microbiome		
-16S rRNA gene sequences from stool samples	315 of 1215 participants of 1000IBD	FASTQ
Microbiome		
-16S rRNA gene sequences from biopsies	107 of 1215 participants of 1000IBD	FASTQ
Microbiome		
-Whole genome shotgun metagenomics sequences from stool samples	355 of 1215 participants of 1000IBD	FASTQ

### Finding and exploring 1000IBD data

The 1000IBD Project can be found in the European Genome-Phenome Archive https://ega-archive.org/studies/EGAS00001002702, and will be added to the local research data catalogue of the UMCG and the University of Groningen, the national biomedical research data catalogue BBMRI-NL (Biobanking and BioMolecular resources Research Infrastructure The Netherlands) (www.bbmri.nl) and the European biomedical research data catalogue BBMRI-ERIC [38] (Biobanking And BioMolecular Resources Research Infrastructure - European Research Infrastructure Consortium). The 1000IBD data can be explored online on our website: https://1000IBD.org. Here, summary statistics of the phenotypes of 1000IBD participants for the entire cohort and for the subsets of participants for whom *omics* data is available can be viewed.

### Downloading 1000IBD research data

The 1000IBD research data can be downloaded from the EGA at https://ega-archive.org using accession number EGAS00001002702. All raw files (FASTQ, IDAT) from genotyping and sequencing of the genome, transcriptome and microbiome will be available to run through custom pipelines.

### Recalling 1000IBD participants for additional sampling

1000IBD participants are recallable for additional sampling. The possibility to recall patients for additional material is only available to UMCG researchers. However, collaborations in which extra material is collected are possible.

### Coauthorship and citing 1000IBD

The arrangements for publication regarding co-authorship or just citation will depend on the extent of cooperation required by the Groningen team. If IBD participants need to be recalled for additional sampling or if large data processing efforts by the Groningen team are required, co-authorship will be required. However, if a limited amount of data is requested from the EGA, citation of the current manuscript would suffice.

### Privacy and controlled access

To share the molecular and clinical data in a responsible way, we need to maintain patient privacy. It is therefore not possible to publicly share extensive phenotype data, and the publicly available phenotype data will remain limited to a basic phenotype set. 1000IBD data is stored externally in the EGA, but because 1000IBD consists of patient data, a controlled access procedure is in place.

### Preparation of next release

While the first data is released, preparations for the second data release are on-going. The second 1000IBD data release will consist of: i. the complete 544 microbiome WGS metagenomes from stool samples from 544 1000IBD participants and ii. pooled targeted resequencing of UC susceptibility loci.

## Conclusions

The 1000IBD project aims to discover molecular subtypes of IBD and biomarker profiles that capture the clinical heterogeneity of IBD and to prioritise new targets for early stage drug discovery or other interventional strategies by generating extensive *multi-omics* and phenotype data of over 1000 IBD patients. The project is a showcase for FAIR data management following the guiding principles for scientific data management and stewardship (FAIR: Findability, Accessibility, Interoperability, and Reusability) [39, 40]. All 1000IBD data that can be shared without violating patient privacy will be made available to the scientific community. Researchers can reuse the 1000IBD data and perform analyses without the need to set up a cohort, collect samples or perform expensive sequencing. The sharing and reusing of the 1000IBD data will drive IBD research forwards.

## Additional file

Additional file 1: **Table S1.** Information_model. This table contains the information model of the 1000IBD phenotypes. (XLS 98 kb)

## Abbreviations

BBMRI-ERIC: Biobanking And BioMolecular Resources Research Infrastructure - European Research Infrastructure Consortium
BBMRI-NL: Biobanking and BioMolecular resources Research Infrastructure The Netherlands
CD: Crohn’s disease
EHR: Electronic Health Record
GI: Gastrointestinal
IBD: Inflammatory bowel disease
IT: Information technology
UC: Ulcerative colitis
UG: University of Groningen in the Netherlands
UMCG: University Medical Center Groningen in the Netherlands
