# Correction to: The 1000IBD project: multi-omics data of 1000 inflammatory bowel disease patients; data release 1

**DOI:** 10.1186/s12876-019-0938-8

**Published:** 2019-03-27

**Authors:** Floris Imhann, K. J. Van der Velde, R. Barbieri, R. Alberts, M. D. Voskuil, A. Vich Vila, V. Collij, L. M. Spekhorst, K. W. J. Van der Sloot, V. Peters, H. M. Van Dullemen, M. C. Visschedijk, E. A. M. Festen, M. A. Swertz, G. Dijkstra, R. K. Weersma

**Affiliations:** 1 Department of Gastroenterology and Hepatology, University of Groningen and University Medical Center Groningen, PO Box 30.001, 9700RB Groningen, the Netherlands; 2 Department of Genetics, University of Groningen and University Medical Center Groningen, Groningen, the Netherlands

**Correction to: BMC Gastroenterology (2019) 19:5**

**https://doi.org/10.1186/s12876-018-0917-5**

Following publication of the original article [1], the authors reported two names have been erroneously spelled as Festen EAM and Van der Sloot KWJ. The correct names are E.A.M. Festen and K.W.J. van der Sloot.

It has been corrected in the original article as well.

The publisher apologizes for any inconvenience caused by this error.
